# Pressure Flow Analysis in the Assessment of Preswallow Pharyngeal Bolus Presence in Dysphagia

**DOI:** 10.1155/2015/764709

**Published:** 2015-01-29

**Authors:** Lara Ferris, Taher Omari, Margot Selleslagh, Eddy Dejaeger, Jan Tack, Dirk Vanbeckevoort, Nathalie Rommel

**Affiliations:** ^1^Gastroenterology Unit, Child, Youth & Women's Health Service, Adelaide, SA, Australia; ^2^School of Medicine, Flinders University, Adelaide, SA, Australia; ^3^Translational Research Center for Gastrointestinal Disorders, KU Leuven, Leuven, Belgium; ^4^ExpORL, Department of Neurosciences, KU Leuven, Leuven, Belgium; ^5^Geriatric Medicine, University Hospital Leuven, Leuven, Belgium; ^6^Center for Swallowing Disorders, University Hospital Leuven, Leuven, Belgium; ^7^Radiology, University Hospital Leuven, Leuven, Belgium

## Abstract

*Objectives*. Preswallow pharyngeal bolus presence is evident in patients with oropharyngeal dysphagia. Pressure flow analysis (PFA) using high resolution manometry with impedance (HRMI) with AIMplot software is a method for objective interpretation of pharyngeal and upper esophageal sphincter (UES) pressures and bolus flow patterns during swallowing. This study aimed to observe alterations in PFA metrics in the event of preswallow pharyngeal bolus presence as seen on videofluoroscopy (VFSS). *Methods*. Swallows from 40 broad dysphagia patients and 8 controls were recorded with a HRMI catheter during simultaneous VFSS. Evidence of bolus presence and level reached prior to pharyngeal swallow onset was recorded. AIMPlot software derived automated PFA functional metrics. *Results*. Patients with bolus movement to the pyriform sinuses had a higher SRI, indicating greater swallow dysfunction. Amongst individual metrics, TNadImp to PeakP was shorter and flow interval longer in patient groups compared to controls. A higher pharyngeal mean impedance and UES mean impedance differentiated the two patient groups. *Conclusions*. This pilot study identifies specific altered PFA metrics in patients demonstrating preswallow pharyngeal bolus presence to the pyriform sinuses. PFA metrics may be used to guide diagnosis and treatment of patients with oropharyngeal dysphagia and track changes in swallow function over time.

## 1. Introduction

Preswallow pharyngeal bolus presence is viewed on videofluoroscopic swallow study (VFSS) or fiberoptic endoscopic examination of swallowing (FEES) amongst many patients presenting with oropharyngeal dysphagia. There are two main causes for this presentation: poor oral bolus containment with premature bolus spillage and/or a delayed pharyngeal swallow trigger. Poor oral bolus containment results in passive or ineffective movement of a liquid or viscous bolus from the oral cavity into the pharynx prior to pharyngeal swallow onset [1]. By definition this occurs whilst the oral preparatory or oral stage of swallow is still underway [1], that is, before or during lingual propulsion. Separately, a delay in the pharyngeal swallow trigger is defined by a failure of a coordinated and timely pharyngeal response following a purposeful transfer of the bolus into the pharynx [2]. Poor oral containment leading to premature bolus spillage can occur in isolation or in combination with a delayed pharyngeal swallow trigger [1–3]. Preswallow pharyngeal bolus presence puts a patient at risk of aspiration.

High resolution manometry with impedance (HRMI) with automated Pressure Flow Analysis (PFA) is a new method to diagnostically interpret pharyngeal and UES function. Pressure sensors detect activity of swallow musculature whilst impedance electrodes provide measures which indicate bolus flow. PFA derives a range of swallow metrics that indicate bolus flow timing, intrabolus pressure, contractile vigour, bolus presence, and UES luminal diameter, making it possible to measure and describe the function of different mechanical components of pharyngeal swallowing. A global swallow risk index (SRI) generated from PFA metrics as a means to amplify dysfunction has shown to correlate with the presence of aspiration and/or postswallow residue as seen on videofluoroscopy [4, 5]. Following on from this previous work, the purpose of this study was to use HRMI in combination with automated PFA to objectively describe pressure-flow patterns in the pharynx and UES in the event of* preswallow* pharyngeal bolus presence as seen on videofluoroscopy. We hypothesised that specific PFA metrics would be altered in patients with preswallow pharyngeal bolus presence compared to patients without preswallow pharyngeal bolus presence and controls. The aim of this study was to identify the altered PFA metrics which may provide a means to describe functional changes in the pharynx in the event of preswallow pharyngeal bolus presence.

## 2. Materials and Methods

### 2.1. Subjects

We analysed VFSS investigations performed in 40 adult patients with broad dysphagia (24 males, mean age 46 yrs, and age range 23–95 yrs) and 8 adult controls (3 males, mean age 38 yrs, and age range 24–47 yrs). At the time of initial investigation, all subjects were enrolled in study protocols that were approved by the Research Ethics Committee, University Hospital Leuven, Belgium. After understanding the study information, all subjects gave their consent freely. In patients with dysphagia, underlying diseases/conditions were identified through a review of medical records. Eighteen patients had a neurological history (10 patients were post stroke, 2 had Parkinson's disease, 1 Huntington's disease, 1 Multiple Sclerosis, 2 Dementia, 1 Spina Bifida and 1 post-neurosurgery). Of the remaining four patients 1 was post-cervical surgery, 1 Wegener disease, 1 postseptic shock, and 1 Diabetes. This database of patients with broad dysphagia has previously been reported on [4–9], and the diverse clinical presentations and dysphagia severity have been purposeful in order to concept-test PFA metrics in relation to radiological measures from VFSS. Previously this has included aspiration status, postswallow residue, UES opening, and in this case preswallow pharyngeal bolus presence.

### 2.2. Measurement Protocol

Studies were performed in the Radiology Department, University Hospital Leuven, with a 3.2 mm diameter solid state high resolution manometry and impedance catheter incorporating 25 1 cm-spaced pressure sensors and 12 adjoining impedance segments, each of 2 cm (Unisensor AG catheter, Attikon Switzerland). Subjects were intubated after topical anaesthesia (lignocaine spray) and the catheter was positioned with sensors straddling the entire pharyngoesophageal segment (velopharynx to proximal esophagus). Pressure and impedance data were acquired at 20 Hz (Solar GI acquisition system, MMS, Netherlands) with the subject sitting upright. Most subjects were tested with at least 5 boluses in the lateral view: liquid (x3), semisolid (x1), and solid boluses (x1). A standard liquid contrast material (MicropaqueH) was given as liquid bolus and used with thickener (Thick & Easy) for semisolid boluses. A low osmotic hydrosoluble Iodium compound (UltravistH) was used when aspiration was suspected. The viscosity of the administered boluses was determined by a Rheomat 115 Viscometer. The Bingham viscosity of the liquid barium (MicropaqueH) was 0.22 Pascal seconds (PAs), 4.50 PAs for the semisolid bolus. All controls were given boluses of 10 mL volume while patients were given either 5 mL or 10 mL volumes as determined on clinical grounds by the attending specialist. Solid boluses consisted of a 4 cm^2^ piece of bread soaked in the appropriate radiological marker which was chewed and swallowed. All swallows were prompted and the first swallow following bolus administration was marked for analysis. All bolus stock contained NaCl to enhance bolus conductivity, improving the impedance measurement.

### 2.3. Videofluoroscopic Assessments

Continuous videofluoroscopy sequences (25 frames/sec) of swallows were analysed by a speech pathologist (Author Lara Ferris) who was not present during acquisition and was blinded to study functional measures, patient history, and clinical reports. Only primary, lateral view swallows were analysed. Swallows with poor image quality were excluded from analysis. Each primary bolus swallow was reviewed to determine movement of the bolus and the level reached from the oral cavity into the distal pharynx prior to the onset of the pharyngeal swallow, defined by onset of rapid laryngeal excursion [1, 10, 11]. The levels reached were defined as follows: still in the oral cavity, base of tongue, valleculae, or pyriform sinuses. Discussions between authors ensured interrater agreeability before the analysis proceeded.

We applied conservative criteria to define clinically significant bolus presence. Bolus head location within the pyriform sinuses was used as the pathological benchmark.

Based on the results for all the analysed swallows patients were classified as follows:

1. Group 1: patients who never demonstrated bolus movement to the pyriform sinuses prior to swallow onset.
2. Group 2: patients who demonstrated bolus movement to the pyriform sinuses at least once prior to swallow onset.

Note: amongst controls, at swallow onset bolus movement was observed in the mouth or at the base of tongue for all swallows.

### 2.4. Pressure Flow Analysis

**Figure 1:**
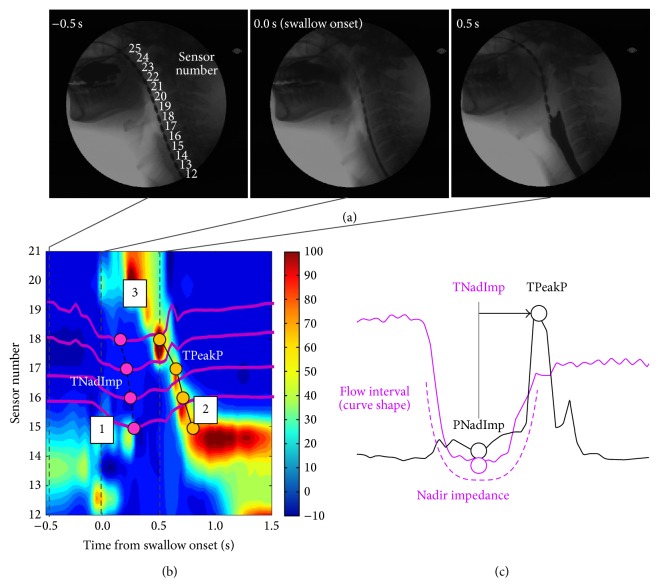
Pharyngeal HRMI and derivation of AIM analysis metrics. (a) Videofluoroscopic images of the catheter in situ in 43-year-old male subject. Consecutive images of a 10-liquid bolus swallow at 0.5 sec before swallow, at swallow onset, and at 0.5 ms after swallow onset. (b) A pressure topography plot of the swallow with impedance waveforms and an example of a PFA metric: PNadImp and PeakP superimposed on the plot. Landmarks defined for AIMplot analysis are marked (1: swallow onset time, 2: position of UES proximal margin post swallow, and 3: position of velopharynx). (c) An illustration showing calculation of the main PFA analysis metrics in the pharynx region. Note: a similar analysis was also applied to the UES region to derive UES Nadir Impedance.

Pharyngeal PFA was performed using automated impedance manometry software (AIMplot) to calculate PFA metrics and two global indices which have been previously validated against VFSS, namely, the SRI, indicative of global dysfunction, and the integrated ratio of nadir impedance to impedance (iZn/Z), indicative of postswallow residue. The calculations used to derive PFA metrics have been previously described [4–9, 12]. In brief, pressure impedance recordings are displayed as pressure topography plots with embedded impedance recordings which show bolus flow movements, the pharyngeal stripping wave, and relaxation and movement of the UES pressure zone (Figure 1(b)). On selection of specific landmarks on the pressure topography space-time plot (with embedded impedance recordings), specific regions of interest (ROI) are mapped. The landmarks identified are (1) time of onset of pharyngeal swallow, (2) position of the UES proximal margin postswallow, and (3) position of the velopharynx during the swallow (numbered 1–3 in Figure 1(b)). There are three ROI encompassing (1) the pharynx, (2) distal pharynx, and (3) UES.

Within each of the ROI, PFA metrics were derived from pressure and impedance waveforms using automated algorithms. Specifically, identification of peak pressure defines the maximum contraction in space and time, the nadir impedance (NadImp) defines the centre of the swallowed bolus in space and time (Figures 1(b) and 1(c)), and the pressure at nadir impedance (PNadImp, Figure 1(c)) defines pharyngeal intrabolus pressure. The time interval from nadir impedance to peak pressure (TNadImp to PeakP, Figure 1(c)) measures the time from bolus passage to pharyngeal contraction; the flow interval (FI) correlates with pharyngeal bolus transit time. The UES nadir impedance (UES NadImp) is measured as a correlate of UES opening diameter [7] and the UES intrabolus pressure (UES IBP) is measured using the established method of Ghosh et al., 2006 [13].

#### 2.4.1. Global Assessment of Pharyngeal Dysfunction

The swallow risk index (SRI) was empirically derived and designed to amplify difference in swallow metrics previously shown to be altered in relation to swallow dysfunction and aspiration risk [4, 5]. These validation studies using concurrent VFSS showed that the SRI for liquid and viscous swallows is significantly higher in patients demonstrating penetration-aspiration compared to patients with no penetration or aspiration [4, 5]. Therefore the SRI quantifies the overall level of swallowing dysfunction potentially predisposing to aspiration risk [4, 5].

#### 2.4.2. Impedance Based Detection of Post Swallow Residue

A postswallow residue score was designed using the integrated ratio of nadir impedance to impedance (iZn/Z ratio) which relates postswallow impedance to the impedance during bolus passage. This measure has shown to be significantly elevated in patients with postswallow residue [8].

### 2.5. Statistical Analyses

Statistical analysis was performed using IBM SPSS Statistics 22. Data gathered from multiple swallows were averaged for each subject. Only liquid boluses (5 or 10 mLs) were assessed. Volume was dependent on clinical decision. Data distribution across the study cohort was nonparametric and therefore medians (interquartile range) are presented. Mann-Whitney *U* tests were used to compare controls and patients grouped on VFSS assessment with Bonferroni correction for multiple comparisons, *P* < 0.017.

## 3. Results

Amongst controls a total of 24 liquid swallows were analysed and 102 swallows were analysed from 40 broad dysphagia patients. Controls showed bolus in the mouth or at the base of tongue at the time of swallow onset in all cases. 20 of 40 patients showed bolus to the pyriform sinuses at least once and this was the most frequent bolus position at swallow onset in 75% of this patient group.

**Figure 2:**
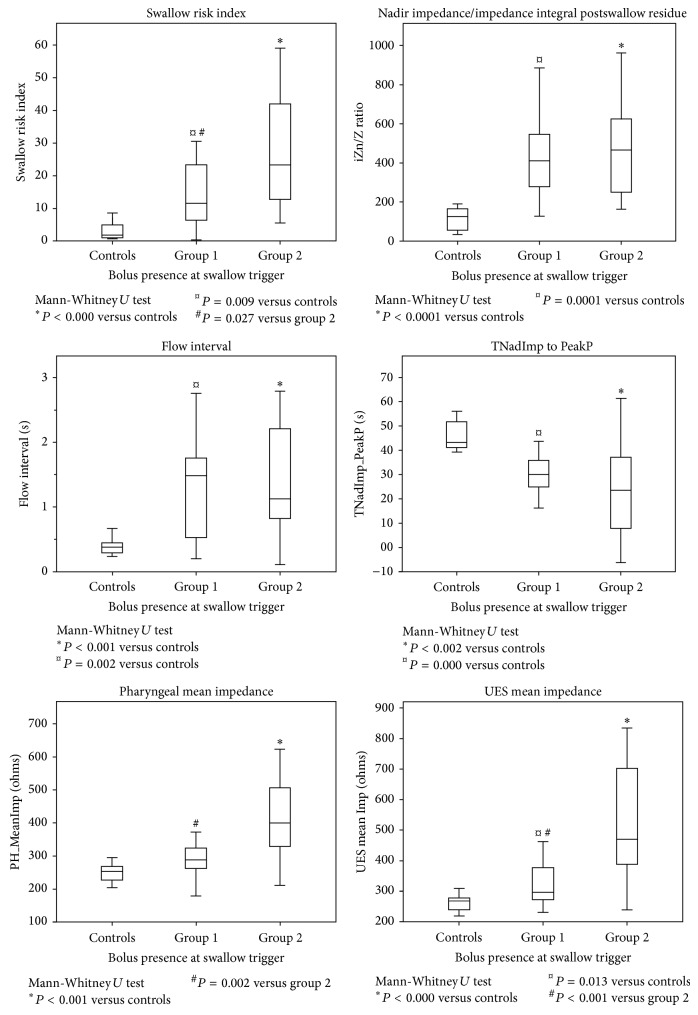
Pressure flow analysis metrics recorded in relation to bolus presence to the pyriform sinuses prior to swallow onset. Group 1: patients NEVER demonstrating bolus to the pyriform sinuses at the time of swallow onset. Group 2: patients demonstrating bolus to the pyriform sinuses AT LEAST ONCE at the time of swallow onset. Controls: in all cases bolus was in the mouth or at base of tongue at time of swallow onset. Data are median (IQR) for liquid swallows. *P* values are from Mann-Whitney *U* tests with Bonferroni correction for multiple comparisons, *P* < 0.017.

As shown in Figure 2 using Mann-Whitney *U* tests, in this cohort of broad dysphagia patients the SRI, a global measure of swallowing dysfunction, was higher in relation to evidence of bolus to the pyriform sinuses prior to the pharyngeal swallow onset compared to controls (*P* < 0.001), and a trend was observed compared to patients who did not spill to the pyriform sinuses (*P* = 0.02).

The TNadImp-PeakP (poor ability for the bolus to be propelled ahead of the pharyngeal stripping wave) was shorter in both patient groups (group 1 *P* < 0.002 and group 2 *P* < 0.001) compared to controls; the flow interval (suggesting extended pharyngeal bolus dwell time) was longer in both patient groups (group 1 *P* < 0.001 and group 2 *P* < 0.002) compared to controls; and the iZn/Z (postswallow residue metric) was significantly higher in both patient groups (group 1 *P* < 0.000 and group 2 *P* < 0.000) compared to controls (Figure 2).

Two individual metrics in this data differentiate the patient groups: group 1: patients without preswallow bolus presence to the pyriform sinuses prior to swallow onset and group 2: patients with preswallow bolus presence to the pyriform sinuses prior to swallow onset. Pharyngeal mean impedance (correlating with reduced pharyngeal distension) was significantly higher (*P* < 0.002) in group 2 compared to group 1 and controls (*P* < 0.001) and the UES mean impedance (correlating with reduced UES opening) was higher (*P* < 0.000) in group 2 compared to group 1 and controls (*P* < 0.001).

## 4. Discussion

Collectively this pattern of pressure-flow differences is consistent with impairment of the mechanisms that drive bolus propulsion, pharyngeal distension, and relaxation and opening of the UES in accordance with laryngeal excursion. Inefficient bolus transfer into the esophagus is the net result.

As has been shown previously, a higher UES impedance recording correlates with reduced UES opening diameter [7] and therefore, in the case of preswallow pharyngeal bolus presence to the pyriform sinuses, an explanation for the increase in pharyngeal and UES mean impedance: bolus movement to the pyriform sinuses before swallow onset leads to a greater loss of bolus volume of the remaining propelled bolus. A smaller bolus volume for propulsion results in reduced distension of the pharynx and UES during the swallow.

VFSS and FEES are currently the most widely used instrumental swallow assessments; however differential diagnosis of oropharyngeal dysphagia can be difficult based on visualisation alone [10]. The use of pharyngeal manometry for the assessment of dysphagia has its limitations and has been described by Nativ-Zeltzer et al. in 2012 [14]; however the integration of concurrent high resolution manometry with impedance allows for dynamic swallow function assessment, and its potential to assist in the evaluation of swallow function is beginning to emerge, as has been demonstrated in this and a number of other recent studies [4–9, 12].

Within the adult population, there is discussion in the literature regarding normal variability for the presence of the bolus lower in the pharynx prior to swallow onset [15–19]. However, the spillage or propulsion of all or part of the bolus to the pyriform sinuses prior to pharyngeal swallow trigger is a pathological event [1–3, 20] suggesting markedly altered mechanics of bolus transport through the pharynx. The clinical relevance in detecting preswallow pharyngeal bolus presence lies in the fact that recognising the causes for bolus presence in the pharynx prior to pharyngeal swallow onset is important for treatment of oropharyngeal dysphagia. Our findings demonstrate how specific PFA metrics are altered in relation to a general pathological observation of preswallow pharyngeal bolus presence in dysphagia patients.

## 5. Conclusion

This pilot study presents specific pressure flow analysis metrics (using integrated high resolution manometry and impedance) which are significantly associated with a known pathological presentation of oropharyngeal dysphagia, that is, preswallow pharyngeal bolus presence to the pyriform sinuses. These results provide reason to further explore the potential differences in pressure flow analysis metrics that may distinguish the causes for preswallow pharyngeal bolus presence, that is, poor oral containment and/or delayed pharyngeal trigger. This will be the focus for future studies. Pressure flow analysis with AIMplot software deriving metrics and a global measure of dysfunction, the swallow risk index, have the potential to guide diagnosis and treatment of patients with oropharyngeal dysphagia and may be used to track patient changes in swallow function over time.
